# Supplementary material for: Implementation of the new integrated algorithm for diagnosis of drug-resistant tuberculosis in Karnataka State, India: How well are we doing?
Source: PLoS One. 2021 Jan 6;16(1):e0244785. doi: 10.1371/journal.pone.0244785 (PMC7787455; doi:10.1371/journal.pone.0244785)
Supplement: S1 File — (DOCX) [file pone.0244785.s001.docx]

poisson reach i.typehf i.specimen ib3.dist2 i.crifres if (crifres<3 & typehf!=9), irr base vce(robust)

Iteration 0: log pseudolikelihood = -959.32125

Iteration 1: log pseudolikelihood = -959.10557

Iteration 2: log pseudolikelihood = -959.10526

Iteration 3: log pseudolikelihood = -959.10526

Poisson regression Number of obs = 1,640

Wald chi2(13) = 247.58

Prob > chi2 = 0.0000

Log pseudolikelihood = -959.10526 Pseudo R2 = 0.0635

| reach | IRR | Robust Std. Err. | z | P>\|z\| | [95% Conf. Interval] |
| --- | --- | --- | --- | --- | --- |
| typehf |  |  |  |  |  |
| Government HF | 1 | (base) |  |  |  |
| Private HF | 1.036441 | .1121584 | 0.33 | 0.741 | .8383628 1.281319 |
|  |  |  |  |  |  |
| specimen |  |  |  |  |  |
| Sputum | 1 | (base) |  |  |  |
| EP | 2.517946 | .3102783 | 7.49 | 0.000 | 1.977678 3.205806 |
| Not recorded | 1.577181 | .4647181 | 1.55 | 0.122 | .8852662 2.809888 |
|  |  |  |  |  |  |
| dist |  |  |  |  |  |
| CRN | 2.194683 | .3778955 | 4.57 | 0.000 | 1.566052 3.075654 |
| BLC | 1 | (base) |  |  |  |
| BLR | .9434234 | .2262816 | -0.24 | 0.808 | .5895826 1.509624 |
| BLU | 1.008624 | .196243 | 0.04 | 0.965 | .6888351 1.476874 |
| KDG | .9976603 | .3368202 | -0.01 | 0.994 | .5147644 1.933557 |
| KLR | 1.299082 | .4154772 | 0.82 | 0.413 | .694068 2.431482 |
| MDY | 1.37685 | .238022 | 1.85 | 0.064 | .9811512 1.932136 |
| MYS | 3.312142 | .4710654 | 8.42 | 0.000 | 2.506385 4.376934 |
| RMN | 1.109548 | .2593023 | 0.44 | 0.656 | .7018104 1.754174 |
| SHI | 1.069078 | .2100363 | 0.34 | 0.734 | .7274081 1.571233 |
|  |  |  |  |  |  |
| crifres |  |  |  |  |  |
| Rifampicin Sensitive | 1 | (base) |  |  |  |
| Rifampicin Resistant | 1.767755 | .2636385 | 3.82 | 0.000 | 1.319703 2.367926 |
|  |  |  |  |  |  |
| _cons | .1593083 | .0207255 | -14.12 | 0.000 | .1234525 .2055781 |

Note: _cons estimates baseline incidence rate.

. estat gof

Deviance goodness-of-fit = 1030.211

Prob > chi2(1626) = 1.0000

Pearson goodness-of-fit = 1178.525

Prob > chi2(1626) = 1.0000

poisson comp i.typehf i.specimen i.crifres ib2.labcode ib1.irlmicro if (crifres<3 & typehf!=9), irr base vce(robust)

Iteration 0: log pseudolikelihood = -412.4166

Iteration 1: log pseudolikelihood = -311.2871

Iteration 2: log pseudolikelihood = -269.24885

Iteration 3: log pseudolikelihood = -268.08338

Iteration 4: log pseudolikelihood = -268.08068

Iteration 5: log pseudolikelihood = -268.08068

Poisson regression Number of obs = 1,196

Wald chi2(7) = 117.62

Prob > chi2 = 0.0000

Log pseudolikelihood = -268.08068 Pseudo R2 = 0.0871

| comp | IRR | Robust Std. Err. | z | P>\|z\| | [95% Conf. Interval] |
| --- | --- | --- | --- | --- | --- |
| typehf |  |  |  |  |  |
| Government HF | 1 | (base) |  |  |  |
| Private HF | 1.638413 | .4553586 | 1.78 | 0.076 | .9502805 2.824847 |
|  |  |  |  |  |  |
| specimen |  |  |  |  |  |
| Sputum | 1 | (base) |  |  |  |
| EP | .5313225 | .5501751 | -0.61 | 0.541 | .0698158 4.043547 |
| Not recorded | .3476996 | .2498522 | -1.47 | 0.142 | .0850241 1.42189 |
|  |  |  |  |  |  |
| crifres |  |  |  |  |  |
| Rifampicin Sensitive | 1 | (base) |  |  |  |
| Rifampicin Resistant | 8.469378 | 1.980865 | 9.13 | 0.000 | 5.355103 13.39477 |
|  |  |  |  |  |  |
| labcode |  |  |  |  |  |
| NTI Bangalore | .6830189 | .1713373 | -1.52 | 0.129 | .4177402 1.116758 |
| IRL Bangalore | 1 | (base) |  |  |  |
|  |  |  |  |  |  |
| irlmicro |  |  |  |  |  |
| Negative | 2.691347 | .8818434 | 3.02 | 0.003 | 1.416009 5.115326 |
| Positive | 1 | (base) |  |  |  |
| Not recorded | 1.454542 | .827308 | 0.66 | 0.510 | .4770713 4.434748 |
|  |  |  |  |  |  |
| _cons | .0550424 | .0080132 | 19.92 | 0.000 | .0413787 .073218 |

Note: _cons estimates baseline incidence rate.

. estat gof

Deviance goodness-of-fit = 378.1614

Prob > chi2(1188) = 1.0000

Pearson goodness-of-fit = 1122.834

Prob > chi2(1188) = 0.9112
