# Supplementary material for: Implementation of the new integrated algorithm for diagnosis of drug-resistant tuberculosis in Karnataka State, India: How well are we doing?
Source: PLoS One. 2021 Jan 6;16(1):e0244785. doi: 10.1371/journal.pone.0244785 (PMC7787455; doi:10.1371/journal.pone.0244785)
Supplement: Attachment — Submitted filename: Response to reviewers 22 Nov 20.docx [file pone.0244785.s003.docx]

Review Comments to the Author

Please use the space provided to explain your answers to the questions above. You may also include additional comments for the author, including concerns about dual publication, research ethics, or publication ethics. (Please upload your review as an attachment if it exceeds 20,000 characters)

Reviewer #1: Comments: Thank you for the opportunity to review this revised manuscript. The authors have made changes to clarify statements in the paper and fix errors as recommended by the reviewers. However, the authors have declined to address issues raised by reviewers that would allow the reader to properly assess their findings and that would improve the manuscript overall, particularly in the methods, results and discussion.

Major issues:
1. The rationale for including patient-level variables in the model was not included in the methods section as requested. As the manuscript is currently written, it remains unclear to the reader why these variables would confound the association between specimen-specific variables and ‘not reaching’. If they are being used as proxies for something related to the specimen then this information should be included in the methods section. If for example, the authors in their response to reviewers have identified that specimens are inherently different when collected from children and this may be associated with ‘not reaching’, then perhaps use broader age categories that have only 2 or 3 levels (children, adult, older adult age groups).

Ans. We thank the reviewer for the comment. We accept that it is not justifiable to include patient level variables and hence have decided to do away with inclusion of the patient level variables both for non- reach and non-completion tables.

Same for key population. These populations are not mutually exclusive and treating them as discrete populations is likely to create noise in the model and not provide a meaningful result. I suspect there are individuals that are HIV positive, reside in a slum and use tobacco. It is recommended that this variable be aggregated to a simple Yes/No of belonging to a key population. Please include in the methods the justification for including patient variables. It is understandable why key population would be associated with being tested, but once a specimen is collected the reason the specimen wouldn’t make it through the algorithm is not clearly outlined in the manuscript with respect to key population or other patient-centered variables. Please support where possible with references and explanation. While this may be the first such TB study in India there are many studies of other similar algorithms, diseases and/or populations from which to draw information from.

Without clear rationale it will be assumed that this was the data available to the authors and so it was included in the model without any consideration of how to best incorporate it (appropriate levels within variables) and why it should be.

Ans. We accept that key population too constitute patient level data, hence we have decided to exclude this variable too from both the tables of non-reach and non-completion.

2. It was requested that model fitting information be reported. The authors declined to do this.
Key practices of model building and validation, and reporting of results are required for reviewers and readers to assess the findings. It will be necessary to include:

• Standard (ANOVA table) model output, either in the body of the paper or as supplementary materials. All statistics packages produces this as output. A table for each of the final models showing the estimate, SE, df, test statistic and p value for each term in the model.
• Tests of model assumptions (e.g. tested for overdispersion and found…)

Ans. The final model output table will be attached as a supplementary file as suggested.

3. Typically, calculation of RR from a Poisson model should use robust error variance as the standard confidence intervals are not valid. Based on the information available regarding the models it is not clear if this was done. Please ensure the appropriate measure of variation was used and detail what was done in the methods section.

Ans. Yes robust error variance was done. We will attach the final model output sheet and table depicting the same as a supplementary file.

4. The authors should also address limitations of the models in the Discussion (e.g., no examination of interaction terms, possibility of low statistical power for some tests)

Ans. We have not examined any interactions, due to the fewer variables we had and the low statistical power. The same will be mentioned in line nos. 498,499.

Minor:
1. It was requested that the authors indicate if multiple specimens were included for individuals. Information regarding this was provided in the response but not incorporated into the manuscript. Please include this in the methods section to provide the reader with this pertinent info.

Ans. Thanks for this valuable observation. We have mentioned about the same in line number 272 and 273 of the revised manuscript.

2. The number not reaching was not clear based on the figures and clarification of why this is was provided in the response to reviewers but not incorporated into the manuscript. A separate figure or inclusion of another cascade in either figure 2 or 3 would be helpful to make this clear to readers. In addition details regarding these 6 negative/indeterminate should be included prior to the “Overall, out of the total 1660 samples,” otherwise it is not clear why the numbers from the RS and RR do not add to the overall numbers.

Ans. Thanks for the suggestion, the description about the 6-rifampicin resistance reports not available/indeterminate has been mentioned at two appropriate paragraphs, one in line nos. 331,332 and another in line nos. 344,345.
